# Collagen nanofiber reinforced alginate hydrogel tube microbioreactors for cell culture

**DOI:** 10.3389/fbioe.2026.1730541

**Published:** 2026-06-19

**Authors:** Xinran Wu, Yakun Yang, Ying Pan, Yong Wang, Xiaojun Lian, Cheng Dong, Shue Wang, Aijun Wang, Wuqiang Zhu, Wansheng Liu, Yuguo Lei

**Affiliations:** 1 Department of Biomedical Engineering, Pennsylvania State University, University Park, PA, United States; 2 Department of Chemistry, Chemical and Biomedical Engineering, University of New Haven, West Haven, CT, United States; 3 Department of Biomedical Engineering, University of California, Davis, Davis, CA, United States; 4 Center for Surgical Bioengineering, School of Medicine, Department of Surgery, University of California, Davis, Sacramento, CA, United States; 5 Institute for Pediatric Regenerative Medicine, Shriners Hospitals for Children, Sacramento, CA, United States; 6 Department of Cardiovascular Medicine, Physiology and Biomedical Engineering, Center for Regenerative Medicine, Mayo Clinic Arizona, Scottsdale, AZ, United States; 7 Department of Animal Science, Pennsylvania State University, University Park, PA, United States; 8 Huck Institutes of Life Sciences, Pennsylvania State University, University Park, PA, United States

**Keywords:** 3D cell culture system, bioreactor system, cell manufacturing, collagen alginate hydrogel microtubules, large-scale cell production

## Abstract

**Introduction:**

Large-scale production of mammalian cells is pivotal for applications in biotechnology, regenerative medicine, and therapeutic manufacturing. However, current bioreactor technologies face significant technical and economic challenges, including excessive cell aggregation, shear stress-induced cell death, batch-to-batch inconsistencies, and limited scalability. We propose that engineering a cell-friendly microenvironment can enhance culture efficiency. Previously, we developed alginate hydrogel microtubes (AlgTubes) that significantly improved cell density and growth rates; however, AlgTubes lack adhesion sites essential for anchorage-dependent cells and frequently break, causing cell leakage and production inconsistencies.

**Methods:**

To address these limitations, we reinforced AlgTubes with collagen nanofibers, creating collagen-alginate hybrid hydrogel microtubes (ColAlgTubes). Collagen was integrated to form a dense nanofiber network interwoven with the alginate mesh, with the dual aim of enhancing mechanical properties and providing cell adhesion sites. ColAlgTubes were fabricated to maintain cell mass within a 400 μm diameter to ensure efficient nutrient exchange and waste removal.

**Results:**

ColAlgTubes successfully formed a reinforced hybrid hydrogel structure in which the collagen nanofiber network improved the mechanical integrity of the alginate matrix while supplying adhesion sites for anchorage-dependent cells. The optimized 400 μm diameter microenvironment supported high cell viability, rapid proliferation, and exceptional yields of 5 × 10^8^ cells/mL.

**Discussion:**

These results demonstrate that ColAlgTubes overcome the key limitations of AlgTubes by combining structural reinforcement with biological functionality. The improved mechanical properties reduce tube breakage and cell leakage, while collagen-derived adhesion sites broaden compatibility to anchorage-dependent cell types. With their scalability, cost-effectiveness, and high efficiency, ColAlgTubes represent a transformative solution for large-scale mammalian cell production across biotechnology, regenerative medicine, and therapeutic manufacturing.

## Introduction

Human and animal cells are integral to a broad spectrum of biomedical and industrial applications. Stem cells, such as human pluripotent stem cells (hPSCs) including human embryonic stem cells (hESCs) and induced pluripotent stem cells (iPSCs), along with their differentiated progeny, are utilized in the treatment of various diseases and injuries (Lei and Schaffer, 2013). They are also essential for disease modeling, drug screening, and toxicity testing (Lei and Schaffer, 2013). Immune cells, like T cells and natural killer cells, are used in cancer immunotherapy (Chang et al., 2023; Wang et al., 2022; Villanueva, 2020; Vivier et al., 2024; Cappell and Kochenderfer, 2023). Additionally, mammalian cells are crucial for producing recombinant proteins and viruses, which are vital for research and clinical applications (Milone and Doherty, 2018; Wang et al., 2024; Ghaleh et al., 2020). These applications need large quantities of high-quality cells (Lei and Schaffer, 2013). For example, treating a single patient requires approximately 10^9^ cardiomyocytes for myocardial infarction (MI) or 10^9^ β cells for Type 1 diabetes (Kropp et al., 2017). The demand is further amplified by the prevalence of degenerative diseases and organ failure, with 1–2.5 million people in the U.S. affected by Type 1 diabetes and around 8 million by MI (Kropp et al., 2017). Similarly, tissue engineering demands large numbers of cells, with approximately 10^10^ hepatocytes or cardiomyocytes needed to construct an artificial human liver or heart (Badylak et al., 2011). Drug discovery efforts, such as screening a million-compound library, can also require 10^10^ cells per screen (Kropp et al., 2017). Moreover, large quantities of mammalian cells, such as Chinese Hamster Ovary (CHO) cells and Human Embryonic Kidney 293 (HEK293) cells, are essential for producing therapeutic biologics, including monoclonal antibodies, enzymes, and viral particles (Milone and Doherty, 2018; Wang et al., 2024; Ghaleh et al., 2020).

Despite the critical need for efficient and scalable cell production, existing methods remain inadequate, particularly for clinical applications (Lei and Schaffer, 2013; Kropp et al., 2017; Jenkins and Farid, 2015). Two-dimensional (2D) culture systems, such as flasks, are widely used but lack the complexity of natural cellular environments. They are also resource-intensive, requiring significant labor, space, and reagents, making them impractical for large-scale production. Three-dimensional (3D) suspension culture systems, such as stirred-tank bioreactors, have been developed to improve scalability (Chen et al., 2014a; Chen et al., 2014b). However, these systems face significant challenges, particularly uncontrolled cell aggregation. Aggregates exceeding 400 µm in diameter suffer from impaired transport of nutrients, oxygen, and growth factors, leading to slower proliferation, apoptosis, and undesired differentiation. While agitation can mitigate aggregation, it also introduces shear forces, negatively impacting cell survival, growth, and differentiation efficiency (Kropp et al., 2017; Kinney et al., 2011; Fridley et al., 2012). As a result, 3D suspension cultures often exhibit high cell mortality, slow proliferation rates, and low volumetric yields. For instance, hPSCs in stirred-tank bioreactors typically undergo only a four-fold expansion over 4 days, yielding approximately 2.0 × 10^6^ cells/mL, which utilizes just 0.4% of the bioreactor volume (Lei et al., 2014; Steiner et al., 2010; Serra et al., 2012). The complexity of hydrodynamic conditions in stirred-tank bioreactors - such as flow dynamics, shear stress, and chemical gradients - adds further challenges. These factors depend on variables including bioreactor design, medium viscosity, and agitation speed, making precise control difficult (Lei and Schaffer, 2013; Kropp et al., 2017; Jenkins and Farid, 2015; Kinney et al., 2011; Fridley et al., 2012; Lei et al., 2014; Steiner et al., 2010; Serra et al., 2012; Ismadi et al., 2014). The variable hydrodynamic conditions contribute to large cell production variation. For example, in cardiomyocyte production from hPSCs, yields from three ∼100 mL batches of hESCs varied widely, ranging from 40 to 100 million cells, with cardiomyocyte purity fluctuating between 54% and 84% (Kempf et al., 2014; Chen et al., 2015). When using a different hPSC line under the same conditions, yields ranged from 89 to 125 million cells, with purity varying from 28% to 88% (Kempf et al., 2014; Chen et al., 2015). Scaling production from 100 mL to 1000 mL requires extensive re-optimization of agitation rates and culture protocols, underscoring the technical and economic challenges of achieving industrial-scale cell manufacturing (Kempf et al., 2014; Chen et al., 2015). Currently, the largest demonstrated hPSC suspension cultures are limited to volumes of just tens of liters (Kropp et al., 2017; Kempf et al., 2016).

To overcome these limitations, we propose the development of hydrogel tube-based 3D microbioreactors. Our previous studies demonstrated that culturing cells within hollow hydrogel microtubes or microbioreactors made from alginate polymers mitigate many of these challenges. These microtubes prevent excessive cell aggregation, enhance mass transport efficiency, and eliminate hydrodynamic stress, leading to high cell viability, rapid proliferation, and significantly improved volumetric yields. Notably, yields of up to 5 × 10^8^ cells/mL have been achieved (Li et al., 2018a; W et al., 2021; Lin et al., 2018a; Wang and Lei, 2019; Lin et al., 2019a; Lin et al., 2019b; Lin et al., 2018b; Lin et al., 2019c; Liu et al., 2023; Li et al., 2018b). These findings highlight the transformative potential of hydrogel tube microbioreactors for scalable, efficient, and cost-effective cell production (Li et al., 2018a; W et al., 2021; Lin et al., 2018a; Wang and Lei, 2019; Lin et al., 2019a; Lin et al., 2019b; Lin et al., 2018b; Lin et al., 2019c; Liu et al., 2023; Li et al., 2018b). Despite their advantages, AlgTubes have critical limitations. They are mechanically fragile, with a significant proportion breaking during cell culture, leading to cell leakage and production failures. Additionally, many cells do not grow well in AlgTubes because they cannot adhere to the alginate surface. There is a critical need for new, robust, and adhesive hydrogel tube microbioreactors. This study introduces the fabrication and application of hybrid collagen-alginate microtubes for cell culture. Within these microtubes, collagen proteins form a nanofiber network that interpenetrates the alginate hydrogel mesh. The collagen nanofibers enhance the alginate hydrogel’s structural integrity and serve as anchoring points for adhesion-dependent cell growth. This advanced system will facilitate cost-effective large-scale cell culture for various biomedical and industrial applications.

## Methods

### Collagen extraction from rat tails

Rat tails were harvested and soaked in 70% ethanol for 30 min. The skin was carefully removed using a scalpel and forceps to isolate the tendons, which were then rinsed three times with PBS. To ensure sterilization, the tendons were submerged in 70% ethanol for at least 1 h. After sterilization, they were soaked in 0.02N acetic acid and stirred at 4 °C for 48 h. The resulting viscous mixture was centrifuged at 10,000 rpm for 60 min at 4 °C to remove debris. The supernatant was collected and dialyzed against 0.02 N acetic acid to obtain the collagen stock solution, which was then quantified using a BCA assay (ThermoFisher, #23227) to determine its final concentration for subsequent applications. The extracted collagen stock was stored at 4 °C prior to use.

### Process ColAlgTubes

Rat tail collagen was adjusted to pH 5.0 using a neutralizing solution (Advanced BioMatrix, #5155) and then combined with 80–120 cP alginate in a pH 5.0 NaCl solution to create a collagen-alginate mixture. A custom-built micro-extruder was used to fabricate ColAlgTubes. A 2% 1500 cP methylcellulose (MC, R&D Systems, #HSC001)) solution containing single cells was pumped into the central channel at a rate of 20 μL/min, while a pre-cooled syringe containing the collagen-alginate solution was placed in a homemade ice box and pumped into the side channel at 61 μL/min. The two solutions were co-extruded into a 50 mM HEPES +100 mM CaCl_2_ buffer (pH 7.4), forming ColAlgTubes. After extrusion, the buffer was replaced with a cell culture medium to support cell growth.

### Scanning electron microscope (SEM) imaging

The nanostructures of ColAlgTubes were characterized using a Zeiss SIGMA VP-FESEM. Before imaging, samples were dehydrated through a graded ethanol series (25%, 50%, 70%, 85%, 95%, and 100%), with each step lasting 5 min. Critical point drying was performed using a Leica EM CPD300 Critical Point Dryer. The samples were sputter-coated with a 4.5 nm iridium layer to enhance conductivity using a Leica EM ACE600 Sputter Coater. SEM images were acquired at an accelerating voltage of 5 kV, with a working distance of 6 mm and magnifications ranging from 150x to 5,000x, allowing detailed visualization of the nanofiber structures.

### Collagen and alginate degradation

ColAlgTubes were incubated with 0.2 mg/mL Collagenase P (Sigma, #11213857001) at 37 °C for 15 min to degrade collagen nanofibers. ColAlgTubes were incubated with 0.5 mM EDTA at room temperature for 15 min to dissolve the alginate hydrogel. Following degradation, the samples were prepared for SEM imaging as required.

### Culturing hPSCs in collagen alginate tubes

The hPSCs used in this study were obtained from WiCell Research Institute. For a typical cell culture, 20 µL of cell solution in ColAlgTubes was suspended in 2 mL of Essential 8 (E8) medium supplemented with 10 μM Y-27632 in a 6-well plate. The culture was maintained in an incubator at 37 °C with 5% CO_2_ and 21% O_2_, and the medium was refreshed daily. For cell passaging, the medium was removed, and ColAlgTubes were dissolved using 0.2 mg/mL Collagenase P containing 0.5 mM EDTA for 20 min. The cell mass was collected by centrifugation at 100 *g* for 5 min, followed by treatment with Accutase (Stemcell Technologies, #NC1793126) at 37 °C for 10 min. The cells were then dissociated into single cells for subsequent culture or cryopreservation. The experiments were performed under static culture conditions.

### Cardiomyocyte differentiation

hPSCs in ColAlgTubes were cultured in E8 medium supplemented with 10 μM Y-27632 (LC Laboratories, #Y-5301) until the diameter of the cell aggregates reached the inner diameter of the ColAlgTubes. On Day 0, the medium was replaced with Essential 5 (E5) medium containing 10% lipid mix and 5 μM CHIR9902 (Sigma, SML1046). After 24 h, the medium was replaced with E5 medium containing 10% lipid mix and 3 μg/mL Heparin. From Day 2 to Day 4, the medium was refreshed daily with E5 medium containing 10% lipid mix (Gibco, #3177257), 3 μg/mL Heparin (Sigma, #H3149), and 3 μM IWR1 (Cayman Chemical, #13659). From Day 5 to Day 6, the medium was refreshed daily with E5 medium containing 10% lipid mix and 3 μg/mL Heparin. From Day 7 to Day 10, the medium was refreshed every other day with an E5 medium containing 10% lipid mix and 20 μg/mL insulin. From Day 11 to Day 18, the medium was refreshed every other day with DMEM medium (without glucose, glutamine, and pyruvate) supplemented with 5 mM Lactate. Cardiomyocytes were collected on Day 18 for analysis. For long-term culture, the medium was switched to α-MEM supplemented with 5% FBS and refreshed every other day. The experiments were performed under static culture conditions.

### Staining, flow cytometry, and imaging

Single-cell suspensions were fixed in 4% paraformaldehyde (PFA) at room temperature for 15 min. The fixed cells were then incubated overnight at 4 °C with primary antibodies in PBS containing 0.1% Triton X-100% and 0.5% BSA. After extensive washing, secondary antibodies were added and incubated for 2 h at room temperature. The cells were washed three times with PBS supplemented with 0.5% BSA before analysis using the Attune® NxT™ Acoustic Focusing Cytometer (ThermoFisher). Flow cytometry data were processed using FlowJo software. LIVE/DEAD® Cell Viability staining was performed following the manufacturer’s instructions for cell viability assessment. Phase-contrast and fluorescence imaging were conducted using a Zeiss Axio Observer Fluorescent Microscope.

### Statistical analysis

Data analysis was conducted using GraphPad Prism 8, with results presented as mean ± standard error of the mean. Statistical significance was determined using appropriate tests based on the type of comparison: One-way analysis of variance (ANOVA) was used for multiple group comparisons (three or more groups). Unpaired two-tailed t-tests were performed for comparisons between the two groups. Statistical significance was indicated as follows: p < 0.05 (*), p < 0.01 (**), p < 0.001 (***).

## Results

### Fabrication of ColAlgTubes

**FIGURE 1:**
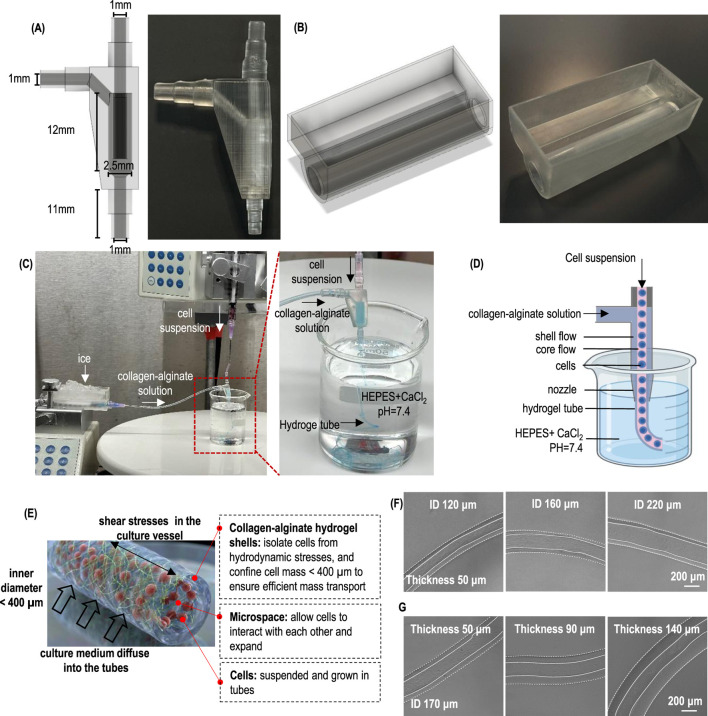
The Collagen-Alginate Hydrogel Tube (ColAlgTube) Microbioreactors. **(A)** Design and printed version of the new micro-extruder. **(B)** Design and printed version of the cooling box used for temperature control. **(C,D)** The setup for processing ColAlgTubes includes two syringe pumps, a custom-made micro-extruder, a cooling box, and a HEPES buffer reservoir. To produce ColAlgTubes, a cell solution and an ice-cold collagen-alginate solution (pH = 5.0) are pumped into the central and side channels of the micro-extruder, respectively, forming coaxial core-shell flows. These flows are extruded into a HEPES-CaCl2 buffer (pH 7.4), which neutralizes the collagen-alginate solution. The shell flow forms a hydrogel tube through collagen fiber formation at pH = 7.4 and alginate crosslinking via Ca2+ ions. **(E)** Cells are cultured within ColAlgTubes suspended in the culture medium inside a vessel. The tubes protect cells from hydrodynamic stress and confine the cell mass to a radial diameter of less than 400 μm, ensuring efficient mass transport of nutrients and waste. These hydrogel tubes create a cell-friendly microenvironment, allowing efficient cell interaction, growth, and diffusion of nutrients through the hydrogel shell. **(F)** The inner diameter (ID) is varied from 120 to 220 μm while maintaining a shell thickness of 50 μm. **(G)** The shell thickness is adjusted from 50 to 140 μm while keeping the ID constant at 170 μm.

This study aimed to develop ColAlgTubes as an innovative cell culture platform. Currently, no technology exists for rapidly fabricating hybrid ColAlgTubes for cell culture applications. To address this gap, we designed a specialized micro-extruder with dual inlets and a single outlet (Figure 1A). The extruder was constructed using a Formula 3B printer and clear resin. Additionally, we designed and 3D-printed a cooling box to maintain the collagen-alginate solution at a low temperature (Figure 1B). The ColAlgTube extrusion setup comprises two syringe pumps, the micro-extruder, the cooling box, and a container filled with HEPES buffer (Figures 1C,D). Syringe 1 is loaded with a room-temperature (RT) cell solution, while Syringe 2 contains an ice-cold collagen-alginate mixture (pH = 5.0). The cooling box features a designated channel to hold Syringe 2 and is packed with ice, ensuring the collagen-alginate solution remains below 4 °C (Figures 1B,C). Maintaining a low temperature and acidic pH prevents premature collagen gelation inside the syringe. To fabricate ColAlgTubes, the two solutions are pumped into inlets 1 and 2, generating coaxial core-shell flows that are extruded into a HEPES buffer solution containing 100 mM CaCl_2_ at 37 °C and pH 7.4 (Figures 1C,D). The core flow carries the cell solution, while the shell flow contains the collagen-alginate mixture. At the microscale, these solutions establish stable laminar flows. The acidic collagen solution in the shell flow is neutralized by the core cell solution and the HEPES buffer, triggering the rapid formation of a collagen nanofiber network. Simultaneously, the alginate in the shell undergoes crosslinking with Ca^2+^ ions, forming a stable alginate network. This is the first time to rapidly process an interpenetrating collagen and alginate tube for cell culture.

### Engineering principles and control ColAlgTube dimensions

Similar to our previously developed AlgTubes, ColAlgTubes overcome the limitations of conventional cell culture techniques by providing a physiologically relevant microenvironment for cell growth (Figure 1E). Cells are cultured within microscale ColAlgTubes, which remain suspended in the culture medium. The hydrogel tubes create free spaces for cell-cell interactions and expansion while protecting cells from hydrodynamic stresses within the culture vessel. Additionally, the tubes restrict cell mass to a radial diameter of less than 400 μm, aligning with human tissue’s diffusion limit, ensuring efficient nutrient exchange and waste removal throughout the culture. Moreover, collagen nanofibers serve as adhesive substrates, supporting the growth of anchor-dependent cells. The dimensions of ColAlgTubes can be tuned by adjusting the ratio of core and shell flows, as well as the diameter of the extruder nozzle. Wall thickness can be controlled by modifying the collagen-alginate shell flow rate, with lower flow rates producing thinner walls. Figure 1F and G showcase ColAlgTubes with varying diameters and wall thicknesses, highlighting the adaptability of this system.

### Nanostructures of ColAlgTubes

**FIGURE 2:**
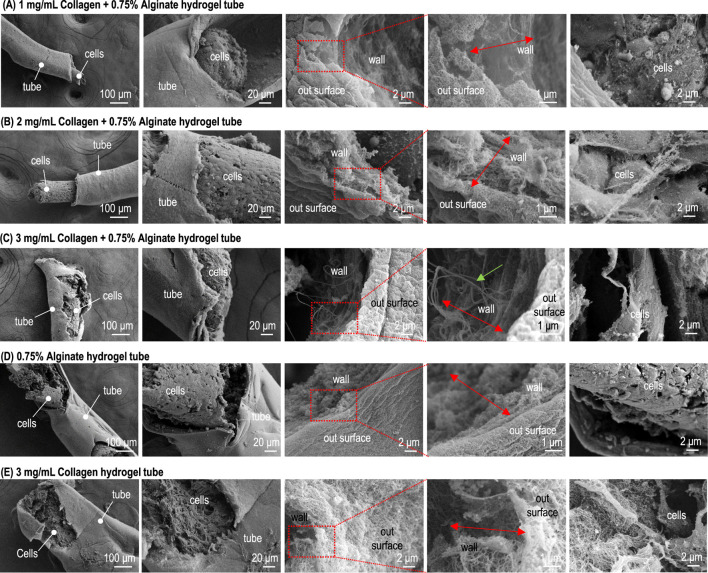
Nanostructures of ColAlgTubes Containing iPSCs. **(A)** 1 mg/mL Collagen +0.75% Alginate hydrogel tube. **(B)** 2 mg/mL Collagen +0.75% Alginate hydrogel tube. **(C)** 3 mg/mL Collagen +0.75% Alginate hydrogel tube. **(D)** 0.75% Alginate-only tube. **(E)** 3 mg/mL Collagen-only tube. The red double-headed arrows indicate the hydrogel tube wall along the radial direction. The nanostructure across the tube wall is uniform along this radial direction. Only the inner surface has a few free collagen nanofibers when 3 mg/mL collagen was used (Green arrow).

We hypothesize that (1) collagen and alginate form an interpenetrating network and (2) collagen nanofibers reinforce the alginate hydrogel, increasing their resistance to force-induced breakage. We used SEM to examine the nanostructures of ColAlgTubes. ColAlgTubes were fabricated with iPSC cells and cultured until the tubes were partially filled. The tubes were then fixed with PFA, dehydrated through a graded ethanol series, metal-sputtered, and imaged using SEM. We captured images of the overall tube structure, inner and outer surfaces, wall nanostructures, and encapsulated cells. The alginate concentration was maintained at 0.75%, while collagen concentrations varied from 1 to 3 mg/mL (Figures 2A–C). For comparison, pure alginate and pure collagen hydrogel tubes were also prepared (Figures 2D,E). In pure alginate hydrogel tubes, a dense mesh-like structure was observed (Figure 2D), while pure collagen hydrogel tubes were composed of collagen nanofibers (Figure 2E). The hybrid ColAlgTubes exhibited nanostructures similar to alginate hydrogel tubes but contained collagen nanofibers interwoven within the dense alginate matrix (Figures 2A–C). Increasing the collagen concentration from 1 mg/mL to 3 mg/mL resulted in more collagen nanofibers. The nanostructures along the tube wall in the radial direction is reatlively uniform.

**FIGURE 3:**
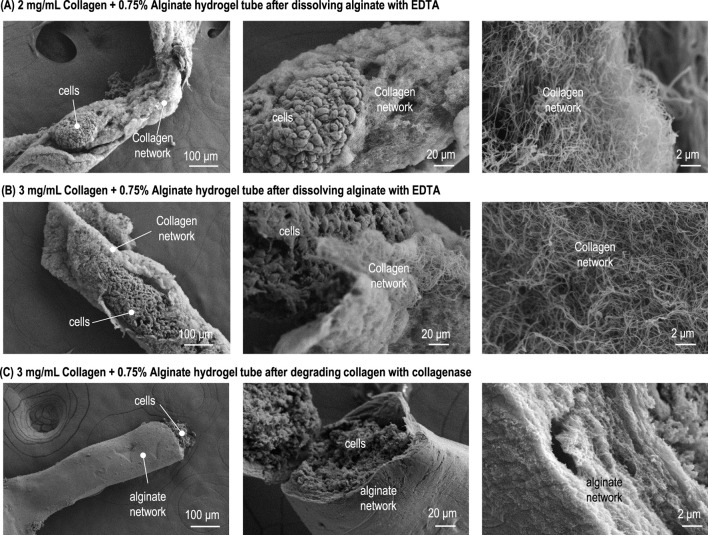
Nanostructures of ColAlgTubes Containing iPSCs. **(A)** 2 mg/mL Collagen +0.75% Alginate hydrogel tube after dissolving alginate with EDTA. **(B)** 3 mg/mL Collagen +0.75% Alginate hydrogel tube after dissolving alginate with EDTA. **(C)** 3 mg/mL Collagen +0.75% Alginate hydrogel tube after degrading collagen with collagenase. The collagen nanofiber network became much thinner after dissolving the alginate.

To further confirm the interpenetrating network, alginate was selectively dissolved using an EDTA solution, allowing clear visualization of the collagen nanofiber network (Figures 3A,B). Conversely, collagen nanofibers were degraded with collagenase, revealing the dense alginate network (Figure 3C). These findings validate our hypothesis that ColAlgTubes consist of an interpenetrating network of collagen nanofibers and alginate hydrogels.

### Culturing HEK293 cells in ColAlgTubes

**FIGURE 4:**
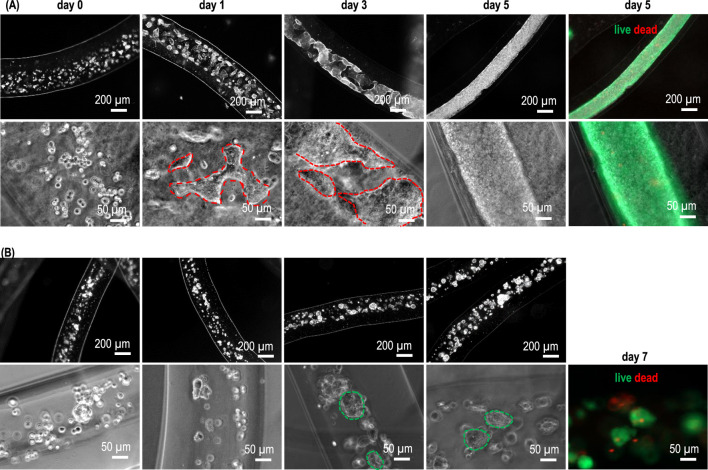
Culturing HEK 293T Cells in ColAlgTubes and AlgTubes. Phase-contrast and Live/Dead staining images of 293T cells cultured in ColAlgTubes **(A)** and AlgTubes **(B)** at various time points. In ColAlgTubes, 293 cells adhered to inner surface of the tubes as outlined with the red dash lines in **(A)**, while in AlgTubes, 293 cells did not attach to the tube and grew as unattached spheroids as outlined with the green dash lines in **(B)**.

To assess if ColAlgTubes can provide adhesion to cells, HEK293 cells (Figure 4A) were processed into the tubes. Within 24 h, the cells adhered to the inner surface and began expanding into colonies. Over time, they proliferated further, eventually filling the entire tube and forming a 3D cell mass. Live/Dead cell staining confirmed that most cells remained viable. Over 4 × 10^8^ cells/mL were successfully harvested. In contrast, when HEK293 cells were cultured in pure AlgTubes, they did not adhere to the tube walls. Instead, they formed unattached spheroids (Figure 4B). These results confirm our hypothesis that incorporating collagen fibers into AlgTubes provides essential anchoring points for cell attachment and growth.

### Culturing hPSCs in ColAlgTubes

**FIGURE 5:**
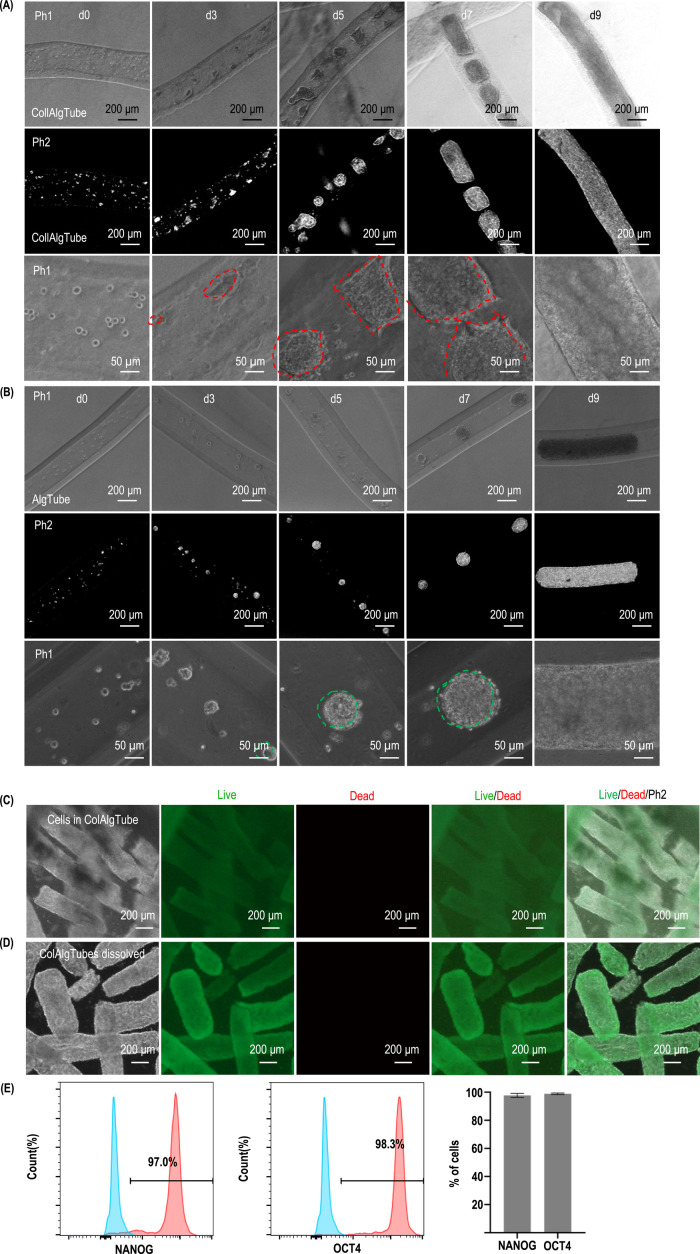
Culturing H9 hESCs in ColAlgTubes. **(A)** Phase-contrast (Ph1) and darkfield (Ph2) images of H9 hESCs cultured in ColAlgTubes on days 0, 1, 3, 5, 7, and 9. Cells adhered to inner surface of ColAlgTubes as shown by the red dash lines. **(B)** Phase-contrast (Ph1) darkfield (Ph2) images of human iPSCs cultured in AlgTubes on days 0, 1, 3, 5, 8 and 9. did not attach to the tube and grew as unattached spheroids as shown by the green dash lines. **(C,D)** Live/Dead staining of day nine H9 cells within ColAlgTubes **(C)** and after release from ColAlgTubes **(C)**. **(E)** Flow cytometry analysis showing that the majority of cells harvested on day 9 express pluripotency markers OCT4 and Nanog.

H9 hESCs were cultured within ColAlgTubes. Within 24 h, the cells adhered to the inner surface of the tubes. By day 3, small colonies had formed, which gradually developed into spheroids by day 5. By day 9, cell proliferation had expanded to fill most of the tube (Figure 5A). Live/Dead staining performed before and after cell release indicated minimal cell death (Figures 5C,D). The dissociated cell mass was subsequently fixed with PFA and stained for the pluripotency markers Nanog and Oct4. Flow cytometry analysis confirmed that 97.0% and 98.3% of the cells expressed Nanog and Oct4, respectively, demonstrating that hPSCs retained their pluripotency after culturing in ColAlgTubes (Figure 5E). Additionally, more than 4.5 × 10^8^ cells per milliliter of microspaces volume were harvested. In contrast, when hPSCs were cultured in pure AlgTubes (Figure 5B), they did not adhere to the tube and grew as unattached spheroids. This further supports the role of collagen fibers in providing anchoring points essential for cell attachment and growth.

### Differentiating hPSCs into Cardiomyocytes in ColAlgTubes

**FIGURE 6:**
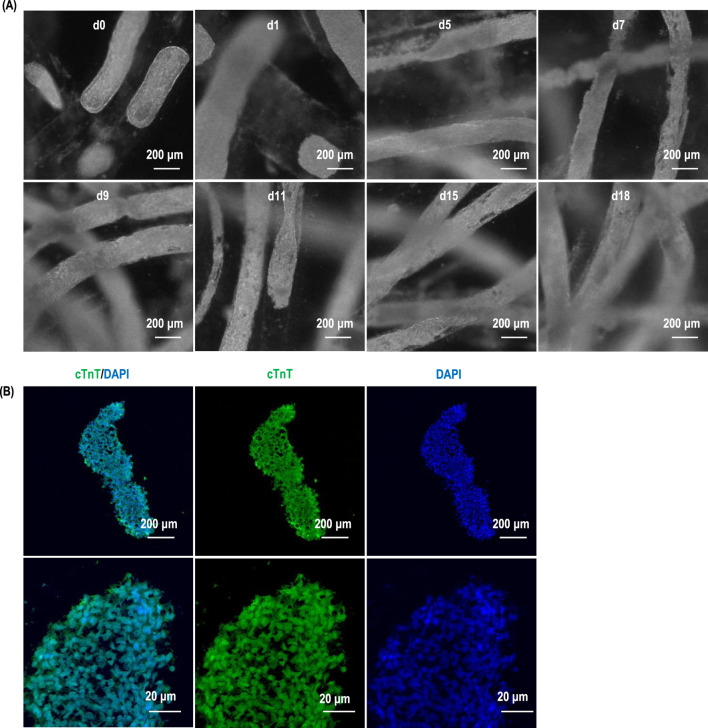
Differentiation of H9 hESCs into Cardiomyocytes in ColAlgTubes. **(A)** Phase-contrast of H9 derived cardiomyocytes cultured in ColAlgTubes at days 0, 1, 5, 7, 9, 11, 15, and 18. **(B)** Immunostaining of cTnT marker for cardiomyocytes differentiated in ColAlgTubes.

To further explore the potential of ColAlgTubes, we differentiated hPSCs into cardiomyocytes. H9 hESCs were cultured in ColAlgTubes and expanded in E8 medium for 7 days. Without requiring passaging, the medium was switched to a mesoderm induction medium to initiate differentiation into mesoderm progenitors. On day 2, the medium was replaced with cardiac progenitor differentiation medium, followed by cardiomyocyte differentiation medium on day 7. From days 11–18, cells were maintained in a metabolic enrichment medium, with the majority remaining viable. Following treatment with EDTA and collagenase, fibrous cardiac tissues were successfully harvested (Figure 6A). Further dissociation of these tissues using Accutase yielded over 3 × 10^8^ cardiomyocytes per milliliter of hydrogel tubes. Immunostaining revealed that most cells expressed the cardiomyocyte marker cTnT, demonstrating that ColAlgTubes effectively support stem cell differentiation (Figure 6B).

### Reduced cell leakage in ColAlgTubes compared to AlgTubes

**FIGURE 7:**
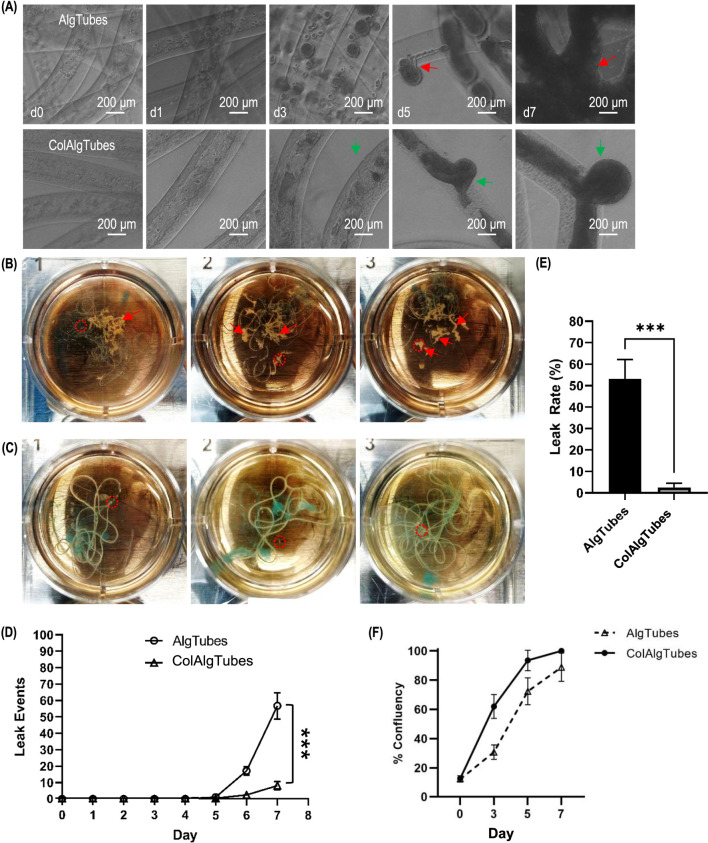
ColAlgTubes Exhibit Fewer Cell Leakage Events Compared to AlgTubes. **(A)** Phase-contrast images of H9 hESCs expanded in AlgTubes and ColAlgTubes for 7 days. Red and green arrows show cell leakage events in AlgTubes and ColAlgTubes, respectively. **(B,C)** Images showing significant cell leakage from AlgTubes, forming large cell aggregates (red arrow) **(B)**, whereas minimal cell leakage is observed from ColAlgTubes **(C)**. **(D)** Quantification of cell leakage events over time for AlgTubes and ColAlgTubes. **(E)** Comparison of leak rates between AlgTubes and ColAlgTubes. Leak rate is defined as the number of cells leaked into the medium divided by the total number of cells in the well. **(F)** Growth curve of H9 hESCs cultured in AlgTubes and ColAlgTubes over 7 days ***: p < 0.001.

Previously, we utilized AlgTubes for cell culture; however, frequent tube breakages were observed (Figure 7A). Since cells did not adhere to AlgTubes, a significant number of cells leaked into the medium through these breaks on days 5, 6, and 7, leading to the formation of large cell aggregates (Figure 7B). In contrast, ColAlgTubes demonstrated significantly greater structural integrity, exhibiting fewer breakages than AlgTubes. Additionally, due to their ability to support cell adhesion, cell aggregates remained attached to the tubes and did not leak into the medium (Figures 7A,C). Quantitative analysis of leakage events (Figure 7D) and the percentage of leaked cells on day 7 (Figure 7E) further confirmed that ColAlgTubes had substantially lower cell leakage than AlgTubes.

In summary, we have demonstrated that ColAlgTubes can be a robust cell expansion and differentiation platform. The interpenetrating collagen-alginate network enhances mechanical stability, significantly reducing cell leakage compared to AlgTubes. Additionally, ColAlgTubes facilitate cell adhesion, making them suitable for culturing both adherent and suspension cells.

## Discussion

Current 2D and 3D cell culture methods face significant challenges in achieving efficient, scalable, and cost-effective large-scale cell production (NCMC, 2016; Baum et al., 2013; National science and technology council, 2016). Key limitations include low cell yields, restricted scalability, high operational costs, and substantial culture variability (NCMC, 2016; Baum et al., 2013; National science and technology council). A fundamental issue is that conventional systems, such as 2D flasks and 3D stirred-tank bioreactors, fail to accurately replicate the complex 3D microenvironments found *in vivo* (NCMC, 2016; Baum et al., 2013; National science and technology council). In their native state, human cells exist within intricate 3D microenvironments that facilitate critical interactions with the extracellular matrix (ECM), support efficient nutrient and oxygen exchange, and minimize hydrodynamic stress (Chen et al., 2014a; Chen et al., 2014b; Thomson et al., 1998; Wong et al., 2010; Kraehenbuehl et al., 2011). We hypothesize that developing advanced culture technologies closely mimicking physiologically relevant microenvironments can overcome these challenges and significantly enhance culture efficiency (Li et al., 2018a; Wang and Lei, 2019).

Hydrogel tube microbioreactors represent a promising approach to creating cell-friendly environments suitable for large-scale culture (Figure 1E) (Li et al., 2018a; Wang and Lei, 2019). We previously developed a method for fabricating hydrogel tubes using alginate polymers (Li et al., 2018a; W et al., 2021; Lin et al., 2018a; Wang and Lei, 2019; Lin et al., 2019a; Lin et al., 2019b; Lin et al., 2018b; Lin et al., 2019c; Liu et al., 2023; Li et al., 2018b). Alginate is abundant, cost-effective, biocompatible, and has an established clinical safety profile. It can be rapidly crosslinked with calcium ions, allowing scalable production of AlgTubes via extrusion. After culture, AlgTubes can be dissolved with EDTA to harvest cells. Their transparency also enables real-time monitoring (Li et al., 2018a; W et al., 2021; Lin et al., 2018a; Wang and Lei, 2019; Lin et al., 2019a; Lin et al., 2019b; Lin et al., 2018b; Lin et al., 2019c; Liu et al., 2023; Li et al., 2018b). Using AlgTubes, we cultured hPSCs with high consistency, maintaining pluripotency and chromosomal stability (Li et al., 2018a). Cultures achieved high viability and rapid expansion (e.g., a 1000-fold increase over 10 days), with volumetric yields of ∼5 × 10^8^ cells/mL - over 200-fold higher than stirred-tank bioreactors. Furthermore, hPSCs differentiated efficiently into endothelial cells (W et al., 2021; Lin et al., 2019a), vascular smooth muscle cells (Lin et al., 2019c), neural stem cells (Lin et al., 2018a), and neurons (Lin et al., 2019b), all reaching yields of 5 × 10^8^ cells/mL. Adult cells, such as T cells, were also successfully cultured in AlgTubes. These findings highlight the transformative potential of hydrogel microtubes for high-efficiency, large-scale production (Wang and Lei, 2019). Despite their success, AlgTubes have limitations. Mammalian cells lack receptors for alginate, preventing adhesion to tube walls, which limits the culture of anchorage-dependent cells like mesenchymal stem cells. Additionally, AlgTubes are mechanically fragile and prone to breakage, leading to cell leakage and variability (Figures 7A,B). These limitations necessitate further advancements in hydrogel tube design for more robust and versatile microbioreactor systems.

Research has shown that incorporating nanofibers into hydrogels is a promising strategy for enhancing mechanical properties and structural integrity (Li et al., 2020; Huang et al., 2020; Gaharwar et al., 2014). Nanofibers, derived from biopolymers such as collagen and silk fibroin or synthetic materials like polycaprolactone (PCL), can form an interpenetrating network within the hydrogel matrix. This structural reinforcement significantly improves the hydrogel’s tensile strength, elasticity, and stability. Additionally, nanofibers enhance cell adhesion, proliferation, and controlled drug release. By integrating nanofibers into hydrogel systems, researchers can develop advanced biomaterials with superior structural integrity and tunable mechanical properties, broadening their applications in regenerative medicine and biotechnology (Li et al., 2020; Huang et al., 2020; Gaharwar et al., 2014). Specific to alginate hydrogels, researchers have explored various nanofibers for reinforcement (Chen et al., 2024). For instance, one study developed laminated composite scaffolds by combining alginate hydrogels with PCL and gelatin electrospun mats (Zare et al., 2021). These scaffolds demonstrated enhanced mechanical properties and controlled biodegradability, making them well-suited for cartilage tissue engineering. Another investigation created a three-dimensional composite scaffold of alginate hydrogels, PCL/gelatin nanofibers, and exosomes, effectively promoting regeneration in rat tympanic membrane perforations (Chahsetareh et al., 2024). A recent review summarized various approaches for developing alginate composite hydrogels (Chen et al., 2024).

However, incorporating nanofibers into alginate hydrogel tubes presents significant challenges compared to embedding nanofibers within bulk hydrogels. To the best of our knowledge, this has not been achieved by others. In this study, we developed an innovative approach to fabricate interpenetrating collagen-alginate hydrogel tubes. By integrating a cooling box, a two-flow micro-extruder, and a buffer exchange system (Figure 1), we successfully established a method for processing collagen-alginate hybrid microtubes. Collagen proteins formed a dense nanofiber network reinforcing AlgTubes (Figures 2, 3). As a result, ColAlgTubes exhibited remarkable durability, with significantly fewer breakages during cell culture, effectively preventing cell leakage (Figure 7). Furthermore, since most cells express receptors for Collagen, they naturally adhered to the ColAlgTubes, enhancing cell attachment and growth (Figures 4, 5).

Our data showed that the cell growth rate, viability, and volumetric yield achieved with ColAlgTubes were comparable to those observed with AlgTubes (Li et al., 2018a). The high growth rate and cell density attainable in this system have significant implications for large-scale cell production. By significantly increasing cell culture density, ColAlgTubes can substantially reduce culture volume, lowering labor requirements, reagent costs, equipment needs, facility space, and manufacturing expenses.

### Future work and limitations

In summary, our work demonstrated that collagen nanofibers can be incorporated into the alginate hydrogel microtubes. The nanofibers can make AlgTubes more resistant to breakage and also provide adhesion points for cells. Similar to AlgTubes, ColAlgTubes also support high-yield cell culture. As advancements in stem cell differentiation protocols continue, future efforts should focus on integrating optimized differentiation processes into ColAlgTubes to enable high-yield production of diverse cell types, such as hPSCs-derived endothelial cells (Lin et al., 2019a), vascular smooth muscle cells (Lin et al., 2019c), red blood cells (Sivalingam et al., 2021; Pavani et al., 2024; Lee et al., 2023), platelets (Thon and Karlsson, 2017; Moreau et al., 2016; Sugimoto et al., 2021; Evans et al., 2021), beta cells, T cells, and neurons (Balboa et al., 2022; Hogrebe et al., 2021; Iriguchi et al., 2021; Montel et al., 2019). Expanding ColAlgTubes applications to include other cell populations, such as adult stem cells and immune cells like T cells, will further increase their utility.

While this study demonstrates the feasibility and functional advantages of ColAlgTubes, several limitations remain that warrant further investigation. First, mechanical properties were evaluated using functional assays rather than quantitative measurements such as tensile strength, modulus, or burst pressure. Second, characterization of the collagen nanofiber network remains primarily qualitative, and more rigorous analysis of fiber size distribution, spatial organization, and reproducibility would strengthen mechanistic understanding. Third, the biological evaluation is limited to a small number of cell types, and broader validation across diverse cell populations, including suspension cells, is needed to establish general applicability. In addition, long-term culture stability, degradation behavior, and performance under large-scale or dynamic bioreactor conditions have not been systematically examined. From a translational perspective, further studies are required to assess immunogenicity, material consistency, and regulatory considerations. Finally, quantitative techno-economic analysis and comprehensive comparisons with other 3D culture systems were beyond the scope of this work. Addressing these areas will be important for future development and for advancing the ColAlgTube platform toward large-scale and practical applications.

## Data Availability

The original contributions presented in the study are included in the article/supplementary material, further inquiries can be directed to the corresponding author.
